# Prognostic importance of systemic inflammation and insulin resistance in patients with cancer: a prospective multicenter study

**DOI:** 10.1186/s12885-022-09752-5

**Published:** 2022-06-25

**Authors:** Guo-Tian Ruan, Hai-Lun Xie, Yi-Zhen Gong, Yi-Zhong Ge, Qi Zhang, Zi-Wen Wang, Xi Zhang, He-Yang Zhang, Meng Tang, Meng-Meng Song, Xiao-Wei Zhang, Ming Yang, Yong-Bing Chen, Kai-Ying Yu, Li Deng, Kun-Hua Wang, Ming-Hua Cong, Han-Ping Shi

**Affiliations:** 1 Department of Gastrointestinal Surgery/Department of Clinical Nutrition, Beijing Shijitan Hospital, Capital Medical University, Beijing, 100038 China; 2 Key Laboratory of Cancer FSMP for State Market Regulation, 10 Tie Yi Road, Beijing, 100038 China; 3 Department of Gastrointestinal Surgery, Guangxi Medical University Cancer Hospital, Nanning, Guangxi Zhuang Autonomous Region, 530021 P.R. China; 4 Guangxi Clinical Research Center for Colorectal Cancer, Nanning, Guangxi Zhuang Autonomous Region, 530021 P.R. China; 5 Yunnan university, Kunming, 650091 China; 6 General surgery clinical medical center of Yunnan province, Kunming, 650032 China; 7 Comprehensive Oncology Department, National Cancer Center/Cancer Hospital, Chinese Academy of Medical Sciences and Peking Union Medical College, Beijing, 100038 China

**Keywords:** Systemic inflammation, C-reactive protein, Insulin resistance, Lipoprotein cholesterol, Overall survival

## Abstract

**Background:**

Systemic inflammation and insulin resistance (IR) are often associated with poor prognosis in cancer. This study aimed to investigate the prognostic value of surrogate systemic inflammation and IR indices in patients with cancer.

**Methods:**

This multicenter prospective study included 5,221 patients with cancer, with a mean age of 59.41±11.15 years, of whom 3,061 (58.6%) were male. The surrogate IR indices included low-density lipoprotein cholesterol to high-density lipoprotein cholesterol (LHR) ratio, total cholesterol to high-density lipoprotein cholesterol (TC/ HDL-c) ratio, triglyceride to high-density lipoprotein cholesterol (TG/HDL-c) ratio, and fasting triglyceride glucose (TyG). Prognostic receiver operator characteristic (ROC) curves and C-indices were used to select a better surrogate IR index in patients with cancer. The prognostic value of the indicators was evaluated using univariate and multivariate survival analyses.

**Results:**

In this study, the median survival time of patients was 44.5 (40.5–51.4) months, and the overall mortality in the 12-month period was 1,115 (53.7%), with 196 mortality events per 1,000 patient-years of patients’ follow-up. The prognostic ROC curve and C-index suggested that the prognostic value of LHR was better than that of the other IR indices. The multivariate-adjusted hazard ratios (HRs) for overall survival (OS) were higher in patients with high C-reactive protein (CRP) (HR, 1.51; 95% confidence interval [CI]: 1.38–1.65) and high LHR (HR, 1.20; 95% CI: 1.06–1.37), respectively. The mortality rate of patients with both high CRP and LHR was 1.75-fold higher than that of patients with both low CRP and LHR.

**Conclusion:**

Both CRP and LHR showed good survival predictions in patients with cancer. CRP combined with LHR can improve the predictive power of patients with cancer.

**Supplementary Information:**

The online version contains supplementary material available at 10.1186/s12885-022-09752-5.

## Background

Inflammation and insulin resistance (IR) play important roles in various chronic diseases, including cancer [1]. Cancer remains a leading cause of death worldwide. According to the Global Cancer Statistics 2020, there were an estimated 19.3 million new cancer diagnoses and 10 million cancer-related deaths in 2020 [2], an increase in both new cancer diagnoses and cancer-related deaths compared to global cancer statistics in 2018 [3]. In many studies, markers of systemic inflammation have been associated with increased cancer risk and mortality [4], including esophagus, gastric, colorectal, liver, pancreas, bladder, and lung cancers [5]. Systemic inflammation can be assessed by various biochemical or blood markers routinely measured in blood tests, as well as by ratios derived from these markers [6]. C-reactive protein (CRP) is a typical acute phase protein, which is produced mainly by liver cells, and its production is a marker of acute regulation of interleukin-6 (IL-6) [7], which rapidly and significantly increases in plasma concentration in response to acute inflammation, infection, and tissue damage [8, 9]. However, circulating levels of CRP can also be moderately elevated during chronic inflammation and cancer [7]. Epidemiological studies have suggested that elevated circulating levels of CRP, as measured with high sensitivity, not only signal an epidemic of cancer, but are also associated with an increased risk of future cancer in apparently healthy people [10, 11].

IR is considered a physiological adaptive response to pregnancy, fasting, exercise, and acute stress environments [12] and is also present in various chronic diseases, such as obesity, type 2 diabetes (T2D), and cancer cachexia [13]. IR in patients with cancer is characterized by increased hepatic glucose production and gluconeogenesis, and unlike T2D, normal fasting glucose is associated with high, normal, or low levels of insulin [14]. This may be due to the redistribution of glucose within tumor cells to maintain energy requirements. Peripheral IR was found in patients with colorectal cancer [15], non-small cell lung cancer (NSCLC) [16], gastrointestinal cancer [17], and mixed malignancies [18] by measuring insulin sensitivity using the gold-standard hyperinsulinemic-euglycemic clamp technique. However, the technique’s invasiveness and high cost limit its applicability. Therefore, to evaluate IR, the homeostasis model assessment of IR (HOMA-IR) has emerged [19]. Although it is used to quantify IR, HOMA-IR is not routinely used in clinical practice. HOMA-IR mainly reflects hepatic insulin resistance and cannot explain the total effect of IR. From a clinical perspective, a simple and easy-to-use predictor of IR could help clinicians identify patients with cancer experiencing IR early and eliminate any additional costs. Previous studies have shown that many novel, indirect, inexpensive, and readily available surrogate markers can adequately predict IR, including low-density lipoprotein cholesterol to high-density lipoprotein cholesterol (LDL-c/HDL-c, LHR) ratio [20], total cholesterol to high-density lipoprotein cholesterol (TC/HDL-c) ratio [21], triglyceride to high-density lipoprotein cholesterol (TG/HDL-c) ratio [21], and fasting triglyceride glucose (TyG) index [21, 22]. Of these indicators, the optimal IR prognostic indicators in patients with cancer are not known.

Chronic IR is found in malignant tumors, but not in benign tumors, and is presumed to contribute to cancer cachexia due to chronic exposure to proinflammatory cytokines, tumor necrosis factor (TNF)-α, IL-6, and insulin growth factor-binding protein [23, 24]. Cytokines may impair insulin signaling by phosphorylating insulin receptors and their substrates [25]. Previous studies have reported that inflammatory responses play an important role in the occurrence of IR through macrophage-mediated innate immune function and T lymphocyte-mediated adaptive immune system function [26]. Because of the close relationship between IR and inflammation, their interaction may help predict mortality in patients with cancer. This study aimed to determine the optimal IR index and investigate the impact of systemic inflammation and IR on total mortality in patients with cancer.

## Methods

### Patient and study design

In this population-based prospective cross-sectional study, information on patients with cancer was recruited from multiple medical centers in China between 2013 and 2021. Patients who met the following criteria were included in the analysis: (I) the pathological diagnosis was cancer, (II) age 18 years or older, and (III) conscious without communication difficulties. There were no strict exclusion criteria. This study was conducted in accordance with the guidelines of the Helsinki Declaration and approved by the Clinical Research Ethics Committee of each participating medical institution (see Additional file 1 ). Prior to the interview, each participant provided written informed consent.

### Anthropometric and laboratory measurements

Through face-to-face interviews, each participant completed a questionnaire that included personal information and medical history. Clinical information was collected from their medical records. Clinical characteristics included age, sex, body mass index (BMI, categoric <18.5; 18.5–24.9; 25–28; >28 kg/m^2^), diabetes (yes/no), hypertension (yes/no), coronary heart disease (yes/no), drinking status (yes/no), alcohol status (yes/no), tumor stage, surgery status (yes/no), radiotherapy status (yes/no), chemotherapy status (yes/no), presence of diabetes (yes/no), nutritional intervention (yes/no), Karnofsky Performance Status (KPS), and tumor type. Tumor stage was defined according to the 8th edition of the TNM system developed by the International Union against Cancer/American Joint Commission on Cancer (UICC/AJCC). All patients underwent anthropometric and routine blood chemistry tests. Anthropometric indicators were measured by clinical staff, and weight and height were measured while the patients wore light clothing and without shoes. BMI was estimated as weight (kg) divided by height (m^2^). Blood or laboratory indicators were collected within 48 h of admission before treatment. Blood samples were collected by trained nurses. After patients fasted overnight (minimum 8 h), blood samples were collected and analyzed in the laboratory. Laboratory markers included CRP, fasting blood glucose (FBG), total cholesterol (TC), triglyceride (TG), high-density lipoprotein cholesterol (HDL-c), and low-density lipoprotein cholesterol (LDL-c). The definition of LHR, TG/HDL-c, and TC/HDL-c were the ratios of LDL-c to HDL-c, TG to HDL-c, and TC to HDL-c, respectively. The TyG index was calculated using the formula: Ln [TC (mg/dl) × FBG (mg/dl)]/2.

### Clinical outcome assessment and patient follow-up

Patients were followed up through outpatient follow-up, inpatient records, and telephone interviews. The primary endpoint was overall survival (OS). OS was calculated from the date of cancer diagnosis to the date of death or the review date of the patient's last follow-up. Patients were followed up until death or June 2021 (the end of follow-up).

### Statistical analysis

R software (version 4.0.3) was used for all statistical analyses. Basic statistics were used to describe patient characteristics. Continuous variables with normal distribution were represented by mean ± standard deviation (SD), and non-normally distributed variables were represented by median and interquartile range (IQR). Categorical variables are expressed as frequencies and percentages (%). The Student’s t-test of independent samples was used for the comparison of normal distribution variables, and the Wilcoxon test was used for the comparison of non-parametric variables. The chi-square test (or Fisher's exact test, when appropriate) was used to compare the distribution of categorical variables. The correlation between prognostic factors was expressed by Pearson's coefficient; the correlation coefficient was >0.4 or <-0.4, and it was considered to have a significant correlation when *P*<0.05. Kaplan–Meier survival analysis using the log-rank test was used to assess OS. To assess hazard ratios (HRs) and 95% confidence intervals (CIs) of OS, a Cox proportional risk model was used. Multivariate survival analysis used different adjustment models to investigate the predictive value of different prognostic indicators. Model 0: unadjusted; Model 1: adjusted for BMI; Model 2: adjusted for age, sex, tumor stage, and BMI; Model 3: adjusted for age, sex, tumor stage, BMI, tumor type, KPS, and surgery, chemotherapy, radiotherapy, smoking, alcohol, and nutritional intervention status; model 4: adjusted for age, sex, tumor stage, BMI, tumor type, KPS, surgery, chemotherapy, radiotherapy, smoking, alcohol, nutritional intervention status, and diabetes. The optimal cutoff values of the prognostic indicators were obtained using the largest selected rank statistic, in which CRP and LHR were 3.96 and 3.56, respectively (see Additional file 2). The prognostic receiver operating characteristic (ROC) curve, Harrell’s concordance index (C-index), and multivariate Cox survival analysis were used to compare the prognostic ability of LHR, TG/HDL-c, TC/HDL-c, and TyG in patients with cancer. All two-tailed *P*-values less than 0.05 were inferred to be statistically significant.

## Results

### Baseline characteristics

In this study, 5,221 patients with cancer were included in the final analysis (see Additional file 3). Demographic and basic characteristics of the patients are shown in Table 1. The mean age of the patients was 59.41±11.15 years, the mean BMI was 22.57±3.51 kg/m^2^, and there were 3,061 (58.6%) male patients. Of the included patients, 616 (11.8%) had a BMI <18.5, 2,899 (55.5%) had a BMI between 18.5 and 23.9, 1,162 (22.3%) had a BMI between 23.9 and 28, and 544 (10.4%) had a BMI ≥28. The median survival time was 44.5 (40.5–51.4) months, and the overall mortality in the 12-month period was 1,115 (53.7%), resulting in 196 mortality events per 1,000 patient-years of patients’ follow-up.

**Table 1:** Baseline characteristics of the study population

Characteristics	Overall Patients	low CRP	High CRP	*P* value	Low LHR	High LHR	*P* value
	(*n*=5221)	(*n*=3565)	(*n*=1656)		(*n*=4634)	(*n*=587)	
Gender (%)							
male	3061 (58.6)	1927 (54.1)	1134 (68.5)	<0.001	2658 (57.4)	403 (68.7)	<0.001
female	2160 (41.4)	1638 (45.9)	522 (31.5)		1976 (42.6)	184 (31.3)	
Age (mean (SD))	59.41 (11.15)	58.63 (11.17)	61.10 (10.93)	<0.001	59.44 (11.07)	59.21 (11.76)	0.63
Age, ≥65 years (%)	1767 (33.8)	1102 (30.9)	665 (40.2)	<0.001	1571 (33.9)	196 (33.4)	0.841
BMI (mean (SD))	22.57 (3.51)	22.81 (3.52)	22.07 (3.44)	0.006	22.51 (3.47)	23.09 (3.77)	<0.001
BMI, kg/m^2^ (%)							
<18.5	616 (11.8)	370 (10.4)	246 (14.9)	0.002	557 (12.0)	59 (10.1)	0.002
18.5-23.9	2899 (55.5)	1942 (54.5)	957 (57.8)		2604 (56.2)	295 (50.3)	
24-27.9	1162 (22.3)	840 (23.6)	322 (19.4)		1005 (21.7)	157 (26.7)	
≥28	544 (10.4)	413 (11.6)	131 (7.9)		468 (10.1)	76 (12.9)	
Diabetes, yes (%)	530 (10.2)	321 (9.0)	209 (12.6)	<0.001	461 (9.9)	69 (11.8)	0.196
Hypertension, yes (%)	1085 (20.8)	685 (19.2)	400 (24.2)	<0.001	935 (20.2)	150 (25.6)	0.003
Coronary heart disease, yes (%)	268 (5.1)	180 (5.0)	88 (5.3)	0.737	237 (5.1)	31 (5.3)	0.942
Smoking, yes (%)	2431 (46.6)	1501 (42.1)	930 (56.2)	<0.001	2112 (45.6)	319 (54.3)	<0.001
Alcohol, yes (%)	1167 (22.4)	743 (20.8)	424 (25.6)	<0.001	1018 (22.0)	149 (25.4)	0.069
Tumor types (%)							
Lung cancer	1776 (34.0)	1103 (30.9)	673 (40.6)	<0.001	1606 (34.7)	170 (29.0)	<0.001
Gastric cancer	766 (14.7)	561 (15.7)	205 (12.4)		689 (14.9)	77 (13.1)	
Esophageal cancer	313 (6.0)	194 (5.4)	119 (7.2)		273 (5.9)	40 (6.8)	
Colorectal cancer	917 (17.6)	679 (19.0)	238 (14.4)		785 (16.9)	132 (22.5)	
Other digestive cancers	394 (7.5)	238 (6.7)	156 (9.4)		342 (7.4)	52 (8.9)	
Breast cancer	464 (8.9)	412 (11.6)	52 (3.1)		437 (9.4)	27 (4.6)	
Female reproductive cancer	177 (3.4)	114 (3.2)	63 (3.8)		159 (3.4)	18 (3.1)	
Urological cancer	132 (2.5)	74 (2.1)	58 (3.5)		112 (2.4)	20 (3.4)	
Nasopharyngeal cancer	126 (2.4)	101 (2.8)	25 (1.5)		108 (2.3)	18 (3.1)	
Other cancer	156 (3.0)	89 (2.5)	67 (4.0)		123 (2.7)	33 (5.6)	
Tumor stage (%)							
I	441 (8.4)	367 (10.3)	74 (4.5)	<0.001	416 (9.0)	25 (4.3)	<0.001
II	943 (18.1)	760 (21.3)	183 (11.1)		872 (18.8)	71 (12.1)	
III	1413 (27.1)	1042 (29.2)	371 (22.4)		1268 (27.4)	145 (24.7)	
IV	2424 (46.4)	1396 (39.2)	1028 (62.1)		2078 (44.8)	346 (58.9)	
Surgery, yes (%)	2596 (49.7)	1980 (55.5)	616 (37.2)	<0.001	2351 (50.7)	245 (41.7)	<0.001
Radiotherapy, yes (%)	581 (11.1)	399 (11.2)	182 (11.0)	0.989	507 (10.9)	74 (12.6)	0.255
Chemotherapy, yes (%)	3312 (63.4)	2281 (64.0)	1031 (62.3)	0.202	2977 (64.2)	335 (57.1)	0.001
Total protein, g/L (mean (SD))	68.63 (6.75)	69.01 (6.31)	67.83 (7.55)	<0.001	68.58 (6.68)	69.03 (7.28)	0.126
Albumin, g/L (mean (SD))	39.04 (5.01)	40.32 (4.42)	36.31 (5.09)	<0.001	39.22 (4.92)	37.64 (5.48)	<0.001
CRP, mg/L (median (IQR))	3.71 (13.20)	3.10 (2.60)	30.80 (46.18)	<0.001	3.44 (10.62)	12.20 (42.46)	<0.001
TC, mmol/L (mean (SD))	4.60 (1.11)	4.68 (1.07)	4.42 (1.19)	<0.001	4.50 (1.02)	5.39 (1.44)	<0.001
Blood glucose, mmol/L (mean (SD))	5.78 (1.76)	5.69 (1.61)	5.97 (2.04)	<0.001	5.73 (1.70)	6.15 (2.14)	<0.001
TG, mmol/L (mean (SD))	1.50 (1.49)	1.53 (1.22)	1.43 (1.94)	0.018	1.45 (1.54)	1.85 (0.99)	<0.001
HDL-c, mmol/L (mean (SD))	1.22 (0.37)	1.28 (0.33)	1.11 (0.43)	<0.001	1.27 (0.37)	0.89 (0.25)	<0.001
LDL-c, mmol/L (mean (SD))	2.82 (0.88)	2.82 (0.83)	2.81 (0.99)	0.617	2.69 (0.76)	3.84 (1.13)	<0.001
LHR (mean (SD))	2.49 (1.30)	2.34 (0.98)	2.81 (1.75)	<0.001	2.22 (0.65)	4.67 (2.51)	<0.001
TC/HDL-c (mean (SD))	4.01 (1.60)	3.85 (1.26)	4.35 (2.12)	<0.001	3.69 (0.87)	6.55 (3.10)	<0.001
TG/HDL-c (mean (SD))	1.38 (1.43)	1.34 (1.27)	1.47 (1.72)	0.002	1.25 (1.24)	2.41 (2.19)	<0.001
TyG (mean (SD))	3.88 (0.29)	3.89 (0.29)	3.86 (0.29)	0.012	7.03 (0.58)	7.36 (0.58)	<0.001
KPS (mean (SD))	85.40 (12.35)	87.31 (10.37)	81.27 (15.00)	<0.001	85.97 (11.87)	80.87 (14.92)	<0.001
KPS, <60 (%)	307 (5.9)	123 (3.5)	184 (11.1)	<0.001	242 (5.2)	65 (11.1)	<0.001
Nutritional intervention, yes (%)	1000 (19.2)	610 (17.1)	390 (23.6)	<0.001	865 (18.7)	135 (23.0)	0.014

*Notes*: *SD* standard deviation, *IQR* interquartile range, *BMI* body mass index, *TC* total cholesterol, *TG* triglyceride, *CRP* C-reactive protein, *LHR* LDL-c/HDL-c ratio, *HDL-c* high-density lipoprotein cholesterol, *LDL-c* low-density lipoprotein cholesterol, *TyG* triglyceride-glucose index, *KPS* karnofsky performance status

### Association and comparison of indices

**Fig. 1:**
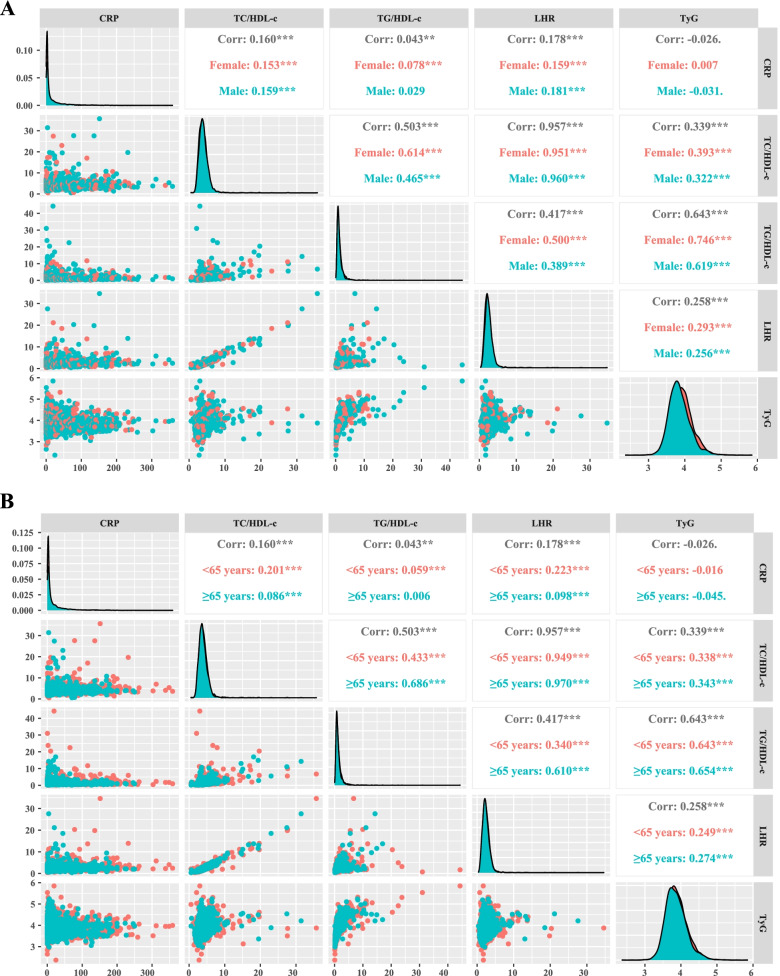
Pearson correlation analysis among CRP, LHR, TC/HDL, TG/HDL, and TyG. **A** Stratified by sex; **B** Stratified by age. Notes: TC: total cholesterol; TG: triglyceride; CRP: C-reactive protein; LHR: LDL-c/HDL-c ratio; HDL-c: high-density lipoprotein cholesterol; LDL-c: low-density lipoprotein cholesterol; TyG: triglyceride-glucose index

**Fig. 2:**
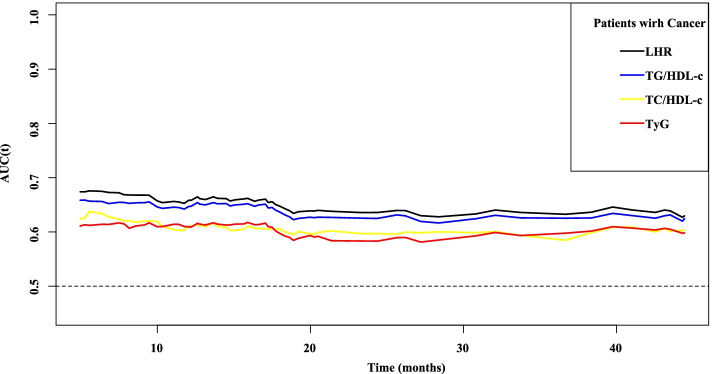
The prognostic AUC of IR makers. Notes: AUC: area under the curve; LHR: LDL-c/HDL-c ratio; HDL-c: high-density lipoprotein cholesterol; LDL-c: low-density lipoprotein cholesterol; TyG: triglyceride-glucose index

Figure 1 shows the correlation between CRP and the different IR indicators, including LHR, TG/HDL-c, TC/HDL-c, and TyG. Pearson correlation analysis showed no significant correlation between CRP levels and the IR indicators. Similarly, CRP and the IR indices showed no correlation in males and females aged ≥65 years and < 65 years. We compared the prognostic value of LHR, TG/HDL-c, TC/HDL-c, and TyG in patients with cancer. First, we compared the predictive ability of the different indicators using ROC analysis for the prognosis of patients with cancer, and the results showed that the area under the curve (AUC) of LHR was higher than that of TG/HDL-c, TC/HDL-c, and TyG (Fig. 2). In addition, C-index results showed that LHR (C-index=0.528, 95% CI=0.514–0.541) was superior to TG/HDL-c (C-index=0.494, 95% CI=0.481–0.507, *vs.* LHR, *P*=0.002), TC/HDL-c (C-index=0.526, 95% CI=0.513–0.540, *vs.* LHR, *P*=0.507), and TyG (C-index=0.513, 95% CI=0.500–0.527, *vs.* LHR, *P*=0.152). Combining the results of the AUC and C-index analyses, we selected LHR as a better predictor of IR in patients with cancer.

### Baseline characteristics and distribution of CRP and LHR

The baseline characteristics classified by CRP showed significant differences in terms of age, sex, tumor stage, and BMI (all *P*<0.05). In addition, the LHR level of patients with high CRP (2.71±1.62) was higher than that of patients with low CRP (2.28±0.83), and the length of stay (*P*<0.001) and cost of hospitalization (*P* =0.585) of patients with high CRP (13.92 days, 26,275.91 Chinese Yuan) was significantly higher than that of patients with low CRP (12.84 days, 25,660.42 Chinese Yuan). The baseline characteristics classified by LHR showed significant differences according to sex, tumor stage, and BMI (all *P*<0.05). In addition, the CRP level of patients with high LHR (34.15±48.70) was higher than that of patients with low LHR (15.62±31.01), and the length of hospital stay (*P*=0.026) and cost (*P* =0.082) of patients with high CRP (14.39 days, 28,718.20 RMB) was significantly higher than that of patients with low CRP (13.24 days, 25,612.84 RMB), respectively Table 1.

**Fig. 3:**
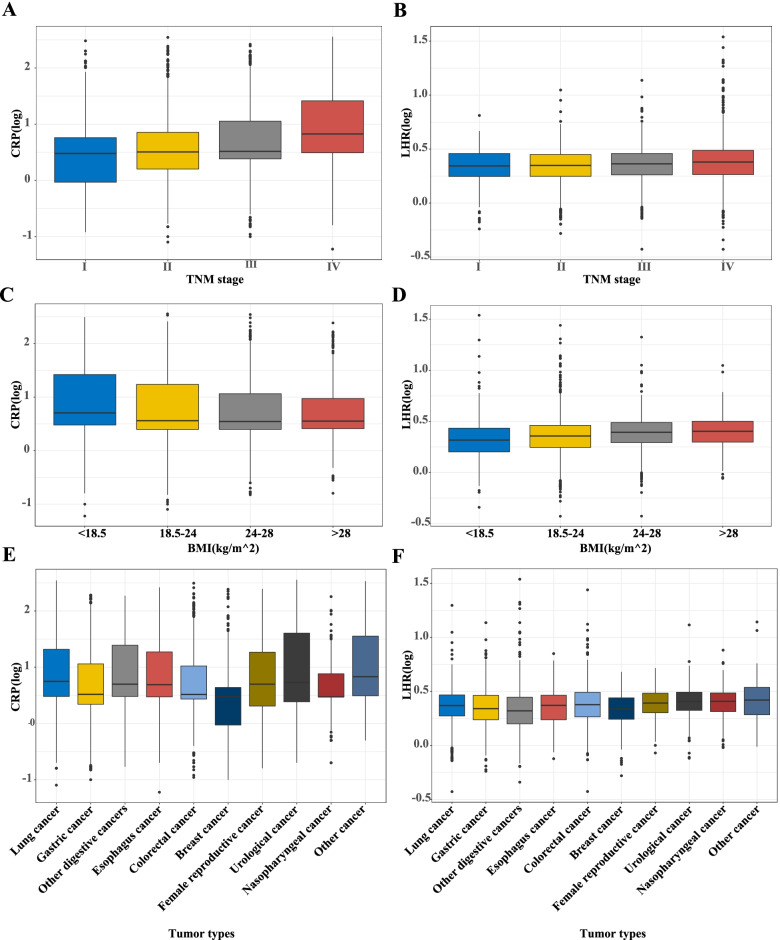
The distribution of CRP and LHR in different groups. **A** CRP in TNM stage groups; **B** LHR in TNM stage groups; **C** CRP in BMI groups; **D** LHR in BMI groups; **E** CRP in tumor types groups; **F** LHR in tumor types groups; Notes: CRP: C-reactive protein; LHR: LDL-c/HDL-c ratio; HDL-c: high-density lipoprotein cholesterol; LDL-c: low-density lipoprotein cholesterol

The distribution of CRP showed that CRP levels increased in patients with TNM stage progression and with BMI decrease. CRP levels were relatively low in breast cancer patients compared to those in patients with other cancers. The distribution of LHR showed that the level of LHR increased with TNM stage progression and BMI increase. LHR was evenly distributed among the different cancer types (Fig. 3). The sex-related difference in CRP distribution showed that CRP levels were higher in males with different TNM stages, BMI, and tumor types (except for female reproductive cancer) than in females. Sex-related differences in the distribution of LHR showed that male LHR levels were higher than female LHR levels in different TNM stages, BMI, and tumor types (except female reproductive cancer) (see Additional file 4).

### Survival analysis

**Fig. 4:**
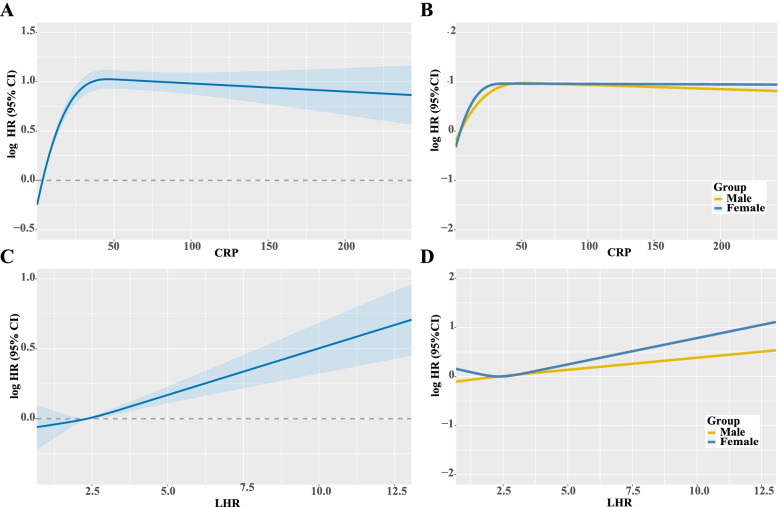
The restricted cubic spline curves of CRP and LHR in patients with cancer. **A** CRP; **B** CRP stratified by sex; **C** LHR; **D** LHR stratified by sex. Notes: CRP: C-reactive protein; LHR: LDL-c/HDL-c ratio; HDL-c: high-density lipoprotein cholesterol; LDL-c: low-density lipoprotein cholesterol

The restricted cubic spline of CRP and LHR indicated that the HRs of patients increased as the levels of CRP and LHR increased. There was no significant difference in HR between male and female patients with CRP, but there was a difference in HR between male and female patients with LHR, and the increasing trend of HR in female patients was greater than that in male patients (Fig. 4).

**Fig. 5:**
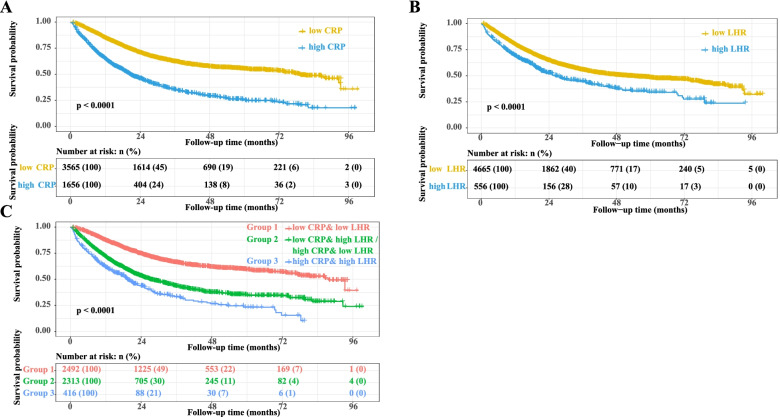
The Kaplan-Meier survival curves. **A** CRP; **B** LHR; **C** CRP combined with LHR. Notes: CRP: C-reactive protein; LHR: LDL-c/HDL-c ratio; HDL-c: high-density lipoprotein cholesterol; LDL-c: low-density lipoprotein cholesterol

The survival curve showed that patients with high CRP and high LHR had a worse prognosis than those with low CRP and low LHR (Fig. 5A-B). CRP showed good survival predictive value in surgical and non-surgical subgroups, radiotherapy and non-radiotherapy subgroups, chemotherapy and non-chemotherapy subgroups, different BMI subgroups, and different tumor stage subgroups (see Additional files 5 and 6). In addition, the subgroup survival curves of LHR showed its survival predictive value (except for the BMI 24–28 subgroup and the tumor stage 1, 2, and 3 subgroups) (see Additional files 7 and 8). Multivariate survival analysis of CRP in patients with cancer showed that the risk of death increased by 13% per SD increase in CRP (95% CI=1.09–1.18). Patients with high CRP had a shorter OS than those with low CRP [model 4: HR (95% CI) =1.60 (1.46–1.75), *P*<0.001]. Patients were divided into four equal groups based on CRP levels (Q1: CRP<2.60, Q2: CRP =2.60–3.71, Q3: CRP =3.71–15.80, and Q4: CRP>15.80), and patients with Q2 [model 4: HR (95% CI) =1.17 (1.01–1.34), *P*=0.031], Q3 [model 4: HR (95% CI) =1.56 (1.37–1.77), *P*<0.001], and Q4 [model 4: HR (95% CI) =1.84 (1.62–2.09), *P*<0.001] had an increased risk of death compared with patients with Q1. The mortality rate increased as CRP levels increased (*P* for trends< 0.001). Multivariate survival analysis of LHR in patients with cancer suggested an increased trend of death risk per SD increase in LHR (HR=1.02, 95% CI=0.98–1.05, *P*=0.332). Patients with high LHR had worse OS than those with low LHR [model 4: HR (95% CI) =1.20 (1.06–1.37), *P*=0.005]. Patients were classified according to LHR into four groups: Q1, LHR<1.81; Q2, LHR =1.81–2.33; Q3: LHR =2.33–2.94; and Q4: LHR >2.94. The prognosis of patients in the Q4 group [model 4: HR (95% CI) =1.14 (1.01–1.29), *P*=0.034] was significantly worse than that in the Q1 group, while there was no significant difference in the prognosis of patients in the Q2 [model 4: HR (95% CI) =0.89 (0.78–1.00), *P*=0.057] and Q3 [model 4: HR (95% CI) =1.03 (0.91–1.16), *P*=0.626] groups compared with that in the Q1 group. However, we also observed an increased risk of death with an increase in LHR (*P* for trend=0.005) (Table 2). Additionally, the sensitivity analysis showed a similar result (see Additional file 9).

**Table 2:** Univariate and multivariate analysis

Variables	OS (model 0) ^a^		OS (model 1) ^b^		OS (model 2) ^c^		OS (model 3) ^d^		OS (model 4) ^e^	
	Crude HR (95%CI)	Crude *P*	Adjusted HR (95%CI)	Adjusted *P*	Adjusted HR (95%CI)	Adjusted *P*	Adjusted HR (95%CI)	Adjusted *P*	Adjusted HR (95%CI)	Adjusted *P*
CRP										
as continues	1.24 (1.20-1.28)	<0.001	1.24 (1.19-1.28)	<0.001	1.17 (1.13-1.21)	<0.001	1.14 (1.09-1.18)	<0.001	1.13 (1.09-1.18)	<0.001
as binary										
By clinical cut-off										
CRP≤10	1		1		1		1		1	
CRP>10	2.35 (2.16-2.56)	<0.001	2.29 (2.10-2.50)	<0.001	1.75 (1.60-1.91)	<0.001	1.52 (1.39-1.65)	<0.001	1.51 (1.38-1.65)	<0.001
as quartile										
Q1(<2.60)	1		1		1		1		1	
Q2(2.60-3.71)	1.30 (1.13-1.49)	<0.001	1.30 (1.13-1.50)	<0.001	1.12 (0.97-1.29)	0.117	1.17 (1.02-1.35)	0.029	1.17 (1.01-1.34)	0.031
Q3 (3.71-15.80)	2.07 (1.82-2.35)	<0.001	2.09 (1.85-2.38)	<0.001	1.66 (1.46-1.88)	<0.001	1.56 (1.37-1.77)	<0.001	1.56 (1.37-1.77)	<0.001
Q4 (>15.80)	3.22 (2.85-3.63)	<0.001	3.13 (2.77-3.54)	<0.001	2.15 (1.90-2.43)	<0.001	1.85 (1.63-2.10)	<0.001	1.84 (1.62-2.09)	<0.001
*p* for trend		<0.001		<0.001		<0.001		<0.001		<0.001
LHR										
as continues	1.10 (1.06-1.15)	<0.001	1.11 (1.07-1.15)	<0.001	1.05 (1.02-1.09)	0.004	1.02 (0.99-1.06)	0.262	1.02 (0.98-1.05)	0.332
as binary										
LHR≤3.56	1		1		1		1		1	
LHR>3.56	1.52 (1.34-1.72)	<0.001	1.59 (1.40-1.80)	<0.001	1.30 (1.14-1.47)	<0.001	1.21 (1.06-1.38)	0.004	1.20 (1.06-1.37)	0.005
as quartile										
Q1(<1.81)	1		1		1		1		1	
Q2(1.81-2.33)	0.88 (0.78-0.99)	0.038	0.93 (0.82-1.05)	0.213	0.94 (0.83-1.06)	0.286	0.89 (0.79-1.01)	0.068	0.89 (0.78-1.00)	0.057
Q3 (2.33-2.94)	1.02 (0.90-1.15)	0.773	1.10 (0.97-1.24)	0.135	1.04 (0.92-1.17)	0.57	1.04 (0.92-1.17)	0.568	1.03 (0.91-1.16)	0.626
Q4 (>2.94)	1.24 (1.10-1.39)	<0.001	1.36 (1.21-1.53)	<0.001	1.20 (1.07-1.36)	0.002	1.15 (1.02-1.30)	0.022	1.14 (1.01-1.29)	0.034
*p* for trend		<0.001		<0.001		0.001		0.003		0.005

*Notes*: *CRP* C-reactive protein, *LHR* LDL-c/HDL-c ratio, *HDL-c* high-density lipoprotein cholesterol, *LDL-c* low-density lipoprotein cholesterol, *HR* hazards ratio, *CI* confidence interval, *BMI* body mass index, *KPS* karnofsky performance status

^a^Model 0: Unadjusted

^b^Model 1: Adjusted for BMI

^c^Model 2: Adjusted for age, sex, BMI and TNM stage

^d^Model 3: Adjusted for age, sex, tumor stage, BMI, tumor types, KPS, surgery, chemotherapy, radiotherapy, smoking, alcohol, and nutritional intervention

^e^Model 4: Adjusted for age, sex, tumor stage, BMI, tumor types, KPS, surgery, chemotherapy, radiotherapy, smoking, alcohol, nutritional intervention, diabetes, hypertension, and coronary heart disease

Multivariate survival analysis of CRP in different tumor types subgroups showed that high CRP in lung [model 4: HR (95% CI) =1.27 (1.11-1.46), *P*=0.001], gastric [model 4: HR (95% CI) =1.37 (1.07-1.75), *P*=0.014], colorectal [model 4: HR (95% CI) =1.37 (1.07-1.75), *P*<0.001], esophageal [model 4: HR (95% CI) =2.42 (1.89-3.10), *P*=0.013], other gastrointestinal [model 4: HR (95% CI) =1.70 (1.24-2.32), *P*=0.001], urological [model 4: HR (95% CI) =3.88 (1.81-8.32), *P*=0.001], nasopharyngeal [model 4: HR (95% CI) =10.53 (2.31-48.11), *P*=0.002], and other cancer types [model 4: HR (95% CI) =2.01 (1.06-3.84), *P*=0.033] predicted worse prognosis (see Additional file 10). Multivariate survival analysis of LHR in different tumor types subgroups showed that high LHR in gastric [model 4: HR (95% CI) =1.48 (1.03-2.13), *P*=0.036] and colorectal [model 4: HR (95% CI) =1.57 (1.17-2.11), *P*=0.003] cancer had worse OS (see Additional file 11).

### Subgroup analysis

**Fig. 6:**
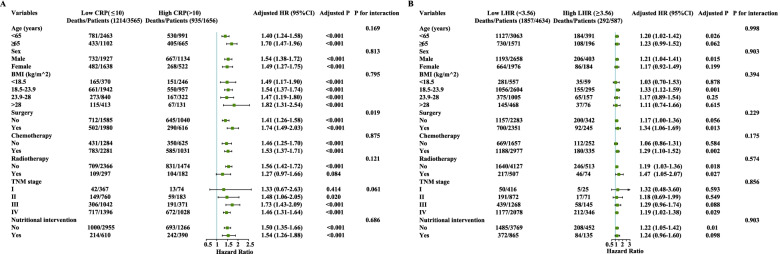
The subgroup analysis of the CRP and LHR in patients with cancer. Adjusted for age, sex, tumor stage, BMI, tumor types, KPS, surgery, chemotherapy, radiotherapy, smoking, alcohol, nutritional intervention, and diabetes. Notes: CRP: C-reactive protein; LHR: LDL-c/HDL-c ratio; HDL-c: high-density lipoprotein cholesterol; LDL-c: low-density lipoprotein cholesterol; HR, hazards ratio; CI, confidence interval; BMI: body mass index; KPS, karnofsky performance status

Subgroup analysis of CRP in patients with cancer showed that CRP levels and surgery status (*P* interaction=0.005) and TNM stage (*P* interaction= 0.031) had significant interactions. However, no significant interaction with CRP was observed in the other subgroups. Subgroup analysis of LHR in patients with cancer showed that no significant interaction with LHR was observed in the subgroups (Fig. 6).

### Combined analysis of CRP and LHR

We investigated the effect of combining CRP and LHR on the prognosis of patients with cancer and found that patients with high CRP and high LHR had a significant increase in HR [model 4: HR (95% CI) =1.77 (1.49–2.05), *P*<0.001] than those with low CRP and low LHR (Fig. 5C, Table 3).

**Table 3:** Combined analysis

Variables	OS (model 0)		OS (model 4)	
	Crude HR (95%CI)	Crude *P*	Adjusted HR (95%CI)	Adjusted *P*
CRP&LHR				
Low CRP & low LHR	1		1	
Low CRP & high LHR or High CRP & low LHR	1.96 (1.79-2.14)	<0.001	1.37 (1.25-1.50)	<0.001
High CRP & high LHR	3.19 (2.74-3.71)	<0.001	1.75 (1.49-2.05)	<0.001
*P* for trend		<0.001		<0.001

*Notes*: *CRP* C-reactive protein, *LHR* LDL-c/HDL-c ratio, *HDL-c* high-density lipoprotein cholesterol, *LDL-c* low-density lipoprotein cholesterol, *HR* hazards ratio, *CI* confidence interval, *BMI* body mass index, *KPS* karnofsky performance status

Model 0: Unadjusted

Model 4: Adjusted for age, sex, tumor stage, BMI, tumor types, KPS, surgery, chemotherapy, radiotherapy, smoking, alcohol, nutritional intervention, diabetes, hypertension, and coronary heart disease

## Discussion

In this study, we found that the prognostic value of LHR was better than that of TG/HDL-c, TC/HDL-c, and TyG based on prognostic ROC and C-index analyses. Therefore, we chose LHR as a better surrogate index for IR. Previous studies have shown that LDL-c and HDL-c are closely related to cancer prognosis. In vitro studies have shown that LDL-c induces cancer cell proliferation, migration, and loss of adhesion [27]. LDL-c was thought to be involved in the upregulation of the mevalonate pathway in peripheral tissues and the production of signaling proteins, such as Ras and Rho [28], while HDL-c may participate in the regulation of cell cycle/apoptosis [29] and cytokine production [30].

The distribution curve of CRP and LHR in patients with cancer showed that the levels of CRP and LHR increased with tumor stage progression, suggesting that inflammation may promote the incidence, stage, and progression of tumors [4]. Cancer-related inflammation has been reported to occur in the tumor microenvironment and systemic circulation, and the increase in local immune cell infiltration and systemic inflammatory response in tumors may be important indicators of cancer progression and prognosis [31]. In addition, low-grade chronic inflammation, characterized by a sustained increase in inflammatory cells and proinflammatory mediators, often elevated before cancer diagnosis, may contribute to carcinogenesis [4]. Previous studies have reported that inflammatory responses play an important role in the occurrence of IR through macrophage-mediated innate immune function and T lymphocyte-mediated adaptive immune system function [1]. We hypothesized that inflammation is an initiating factor for IR and that increased inflammation is accompanied by increased insulin levels.

Interestingly, CRP levels were higher and LHR was lower in patients with low BMI (<18.5 kg/m^^2^), whereas inflammation levels decreased and IR increased with increasing BMI. It can be seen that the presence of inflammation has a relatively greater impact than IR. Patients with cancer are known to be prone to cancer cachexia; patients with low BMI have poorer nutritional status, and inflammation is closely related to malnutrition [32]. However, obese patients frequently develop IR, and various tissues exhibit low cellular sensitivity to insulin activity, which may be due to the body's compensatory mechanisms leading to increased compensatory hormone signaling with growth hormone, epinephrine, or glucagon [14]. In addition, it has been reported that severely malnourished or weight-loss patients with cancer have decreased insulin levels [33, 34], and oral and intravenous glucose stimulation induces abnormally low insulin secretion, which correlates with the degree of weight loss [35]. In addition, we found sex-related differences in different tumor stages, BMI, and tumor subtypes, showing that males had higher levels of inflammation and IR than females.

The results of our survival analysis showed that low CRP and low LHR predicted worse prognosis, while higher CRP and LHR levels were associated with worse prognosis. Subgroup analysis of CRP and LHR showed that CRP had a significant interaction with surgery status and TNM stage. CRP and LHR showed good survival predictive values in patients with cancer. When we combined CRP and LHR, patients with high CRP and high LHR had increased HRs compared with those with low CRP and low LHR. In the study by Natasha et al., patients with IR had mostly low-grade elevated CRP levels (median [IQR] 3.7 [1.8–8.7] mg/L) [36]. In the present study, we found similar results (see Additional file 12). The association between high levels of insulin-like growth factor 1 (IGF-I) and/or insulin and increased risk of various malignancies has been reported [37, 38]. This can be explained by insulin and IGF-I inhibiting apoptosis and enhancing cell proliferation, leading to the accumulation of gene mutations and carcinogenesis [38]. In addition, previous epidemiological studies have shown that CRP is associated with an increased risk of malignancy, anorexia–cachexia syndrome, and poor prognoses, such as tumor recurrence, tumor size, lymph node metastasis, and distant metastasis [39, 40]. The pro-survival effects of TNF-α on cancer cells, IL-6 overstimulation of Janus kinase/signal transducers and transcriptional pathway activators, and matrix metalloproteinases are thought to be mediated [41]. Indeed, there is a link between inflammation and IR, and when a patient has both high inflammation and high IR, the patient's treatment and prognosis are more complicated.

This study was a multicenter prospective cohort study with several major strengths. First, this study compared different IR surrogate markers, selected a sufficient LHR prognostic marker, and proposed cutoff values for the LHR index in patients with cancer. Second, for the first time, we analyzed the combined prognostic impact of CRP and LHR in patients with cancer. Third, we adjusted the multivariate Cox regression analysis using a different adjustment model, which reduced clinical bias, and the results remained robust and reliable. This study had several limitations. First, our study is a cross-sectional study, which only analyzes the blood and biochemical indicators before treatment, and lacks a longitudinal analysis. Second, we selected CRP as an inflammatory marker in patients with cancer. But CRP still has several limitations. For example, secondary conditions, such as infections, might affect CRP levels. Although it shows good predictive performance, this indicator is also a single result and requires multiple measurements and analyses. Considering that China is a country with unbalanced economic development, the measurement of CRP is a detection index with relatively high medical expenses, which may become a factor limiting its applicability. Other inflammatory indicators may be included in future analyses. Third, the IR indicator we used is a simple and readily available surrogate indicator, which is one of our main limitations. Fourth, our study did not collect patient-related medication status, for example, the use of statins may affect blood lipid levels, which in turn affects the prognosis of patients, and relevant data should be added in future studies. Finally, our study involved results from multiple cancer types, and there might be heterogeneity between different cancers, such as the prognostic value of CRP and LHR in patients with early cancer was not as good as in those with advanced cancer, we hypothesized that this was due to the heterogeneity of different cancer types, although these results need further validation.

## Conclusions

This study highlights the importance of systemic inflammation and IR in the prognosis of patients with cancer. The prognostic value of LHR in patients with cancer is better than that of TG/HDL-c, TC/HDL-c, and TyG. In addition, our results suggest that CRP and LHR can predict the survival of patients with cancer. Both high CRP and high LHR predicted poor OS. The mortality rate of patients with both high CRP and LHR was 1.75-fold higher than that of patients with low CRP and low LHR.

## Supplementary Information

**Additional file 1.**

**Additional file 2. **Optimal cut-off value of LHR. Notes: LHR: LDL-c/HDL-c ratio; HDL-c: high-density lipoprotein cholesterol; LDL-c: low-density lipoprotein cholesterol.

**Additional file 3. **Flowchart of patient selection for this study.

**Additional file 4. **The distribution of CRP and LHR stratified by sex in different groups (A) CRP in TNM stage groups; (B) LHR in TNM stage groups; (C) CRP in BMI groups; (D) LHR in BMI groups; (E) CRP in tumor types groups; (F) LHR in tumor types groups; Notes: CRP: C-reactive protein; LHR: LDL-c/HDL-c ratio; HDL-c: high-density lipoprotein cholesterol; LDL-c: low-density lipoprotein cholesterol.

**Additional file 5. **The Kaplan-Meier survival curves of CRP in different subgroups. (A) non-surgery patients; (B) surgery patients; (C) non-chemotherapy patients; (D) chemotherapy patients; (E) non-radiotherapy patients; (F) radiotherapy patients. Notes: CRP: C-reactive protein.

**Additional file 6.** The Kaplan-Meier survival curves of CRP in different subgroups. (A) BMI<18.5; (B) BMI:18.5-24; (C) BMI: 24-28; (D) BMI>28; (E) TNM stage I; (F) TNM stage II; (G) TNM stage III; (H) TNM stage IV. Notes: CRP: C-reactive protein. 

**Additional file 7. **The Kaplan-Meier survival curves of LHR in different subgroups. (A) non-surgery patients; (B) surgery patients; (C) nonchemotherapy patients; (D) chemotherapy patients; (E) non-radiotherapy patients; (F) radiotherapy patients. Notes: LHR: LDL-c/HDL-c ratio; HDL-c: high-density lipoprotein cholesterol; LDL-c: low-density lipoprotein cholesterol.

**Additional file 8. **The Kaplan-Meier survival curves of LHR in different subgroups. (A) BMI<18.5; (B) BMI:18.5-24; (C) BMI: 24-28; (D) BMI>28; (E) TNM stage I; (F) TNM stage II; (G) TNM stage III; (H) TNM stage IV. Notes: CRP: C-reactive protein.

**Additional file 9.**

**Additional file 10.**

**Additional file 11.**

**Additional file 12. **The distribution of CRP between high LHR and low LHR. Notes: CRP: C-reactive protein; LHR: LDL-c/HDL-c ratio; HDL-c: high-density lipoprotein cholesterol; LDL-c: low-density lipoprotein cholesterol.

## Data Availability

The datasets used and/or analysed during the current study available from the corresponding author on reasonable request.
