# Supplementary material for: Prognostic importance of systemic inflammation and insulin resistance in patients with cancer: a prospective multicenter study
Source: BMC Cancer. 2022 Jun 25;22:700. doi: 10.1186/s12885-022-09752-5 (PMC9233357; doi:10.1186/s12885-022-09752-5)
Supplement: Supplementary file 1 — Additional file 1. [file 12885_2022_9752_MOESM1_ESM.pdf]

Supplementary Materials

Additional file 1 List of participating hospitals in this study

Names
Cancer Center of the First Hospital of Jilin University
Daping Hospital, Third Military Medical University
First Affiliated Hospital of Sun yat-sen University
Fujian Cancer Hospital
Beijing Shijitan Hospital
Affiliated Provincial Hospital of Anhui Medical University
Peking University Cancer Hospital and Institute
The First People's Hospital of Foshan
Xingtai People’s Hospital, Hebei Medical University
National Cancer Center/Cancer Hospital, Chinese Academy of Medical Sciences
Tumor Hospital of Yunnan Province
Xijing Hospital, Fourth Military Medical University
Ruijin Hospital, Shanghai Jiao Tong University School of Medicine
The First Affiliated Hospital of Kunming Medical University
Harbin Medical University Cancer Hospital
The Fourth Affiliated Hospital, Harbin Medical University
Affiliated Hospital of Zunyi Medical University
The First Hospital of Hebei Medical University
