# Supplementary figures and images for: Prognostic importance of systemic inflammation and insulin resistance in patients with cancer: a prospective multicenter study
Source: BMC Cancer. 2022 Jun 25;22:700. doi: 10.1186/s12885-022-09752-5 (PMC9233357; doi:10.1186/s12885-022-09752-5)

### Additional file 3

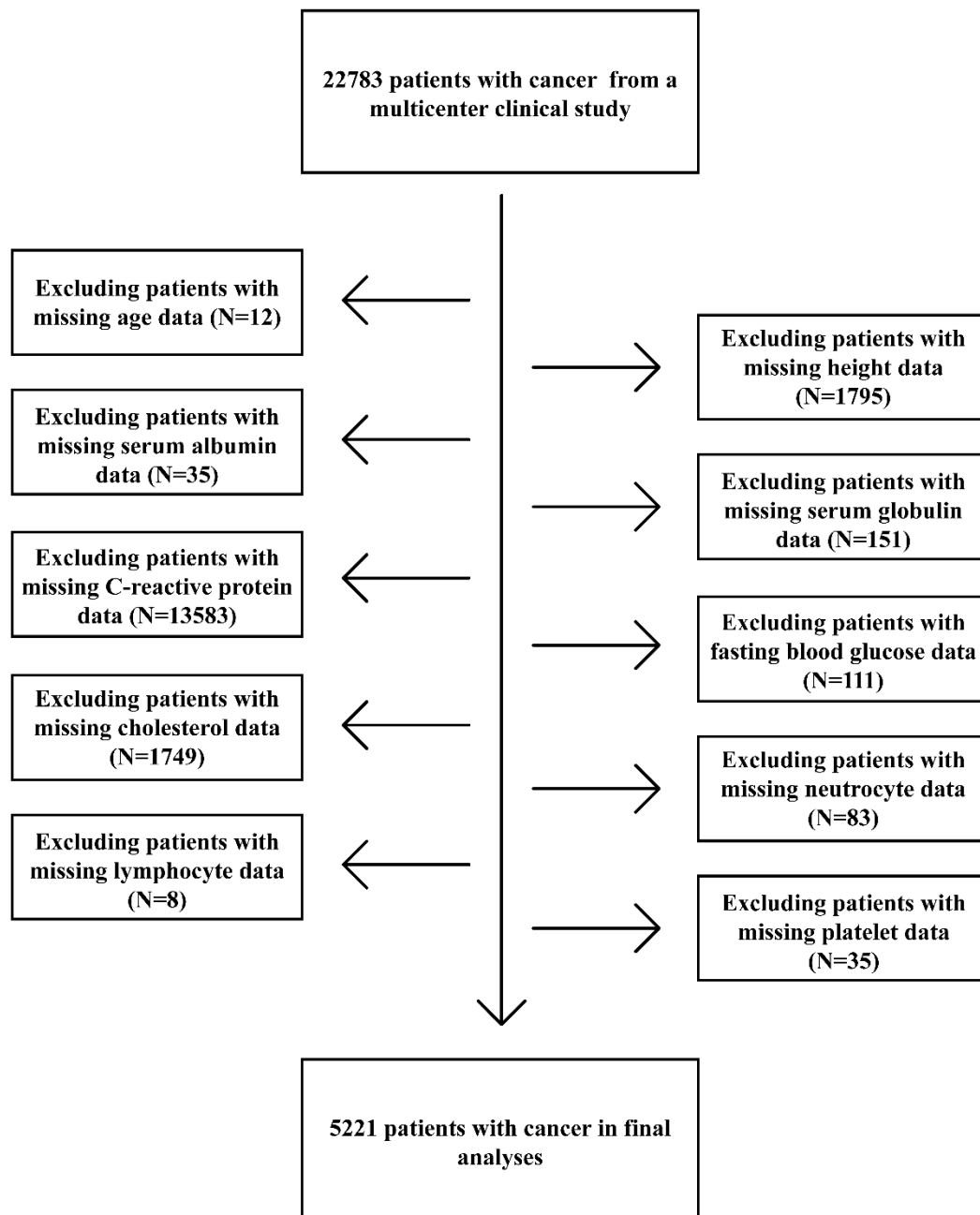

Additional file 3 Flowchart of patient selection for this study.

Supplement: Supplementary file 3 — Additional file 3. Flowchart of patient selection for this study. [file 12885_2022_9752_MOESM3_ESM.pdf]
