# Supplementary material for: Prognostic importance of systemic inflammation and insulin resistance in patients with cancer: a prospective multicenter study
Source: BMC Cancer. 2022 Jun 25;22:700. doi: 10.1186/s12885-022-09752-5 (PMC9233357; doi:10.1186/s12885-022-09752-5)
Supplement: Supplementary file 4 — Additional file 4. The distribution of CRP and LHR stratified by sex in different groups (A) CRP in TNM stage groups; (B) LHR in TNM stage groups; (C) CRP in BMI groups; (D) LHR in BMI groups; (E) CRP in tumor types groups; (F) LHR in tumor types groups; Notes: CRP: C-reactive protein; LHR: LDL-c/HDL-c ratio; HDL-c: high-density lipoprotein cholesterol; LDL-c: low-density lipoprotein cholesterol. [file 12885_2022_9752_MOESM4_ESM.pdf]

Additional file 4

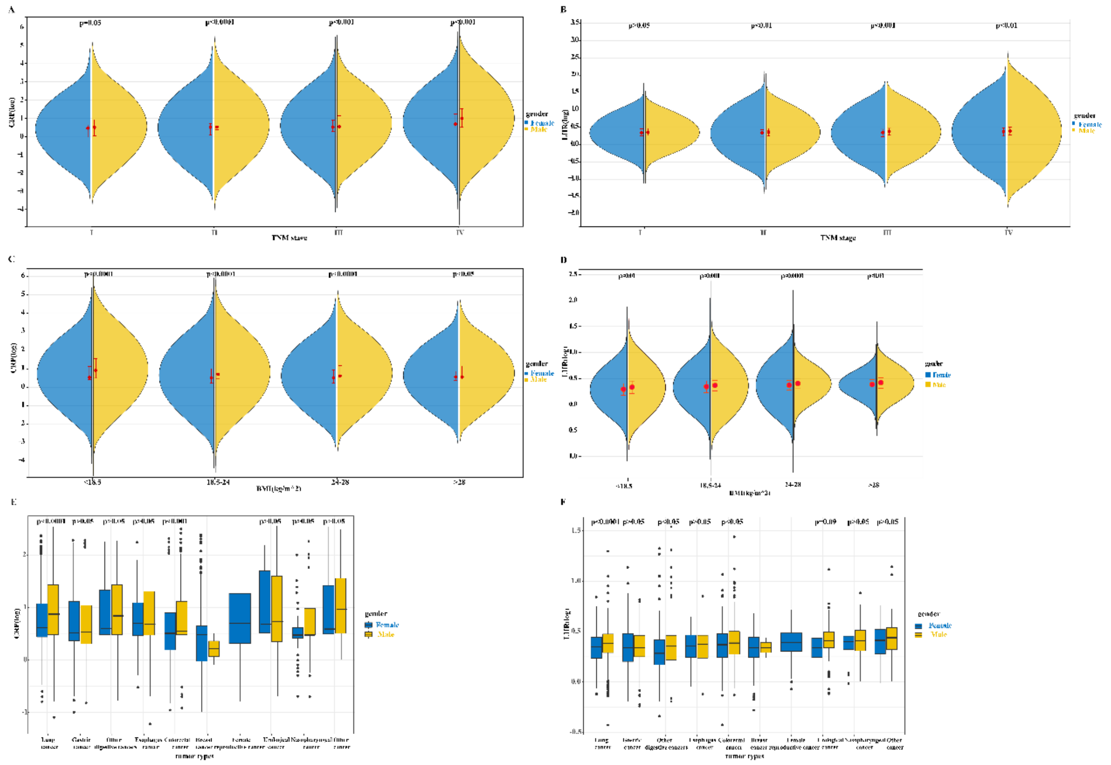

Additional file 4 The distribution of CRP and LHR stratified by sex in different groups

(A) CRP in TNM stage groups; (B) LHR in TNM stage groups; (C) CRP in BMI groups; (D) LHR in BMI groups; (E) CRP in tumor types groups; (F) LHR in tumor types groups;

Notes: CRP: C-reactive protein; LHR: LDL-c/HDL-c ratio; HDL-c: high-density lipoprotein cholesterol; LDL-c: low-density lipoprotein cholesterol.
