# Supplementary material for: Prognostic importance of systemic inflammation and insulin resistance in patients with cancer: a prospective multicenter study
Source: BMC Cancer. 2022 Jun 25;22:700. doi: 10.1186/s12885-022-09752-5 (PMC9233357; doi:10.1186/s12885-022-09752-5)
Supplement: Supplementary file 5 — Additional file 5. The Kaplan-Meier survival curves of CRP in different subgroups. (A) non-surgery patients; (B) surgery patients; (C) non-chemotherapy patients; (D) chemotherapy patients; (E) non-radiotherapy patients; (F) radiotherapy patients. Notes: CRP: C-reactive protein. [file 12885_2022_9752_MOESM5_ESM.pdf]

Additional file 5

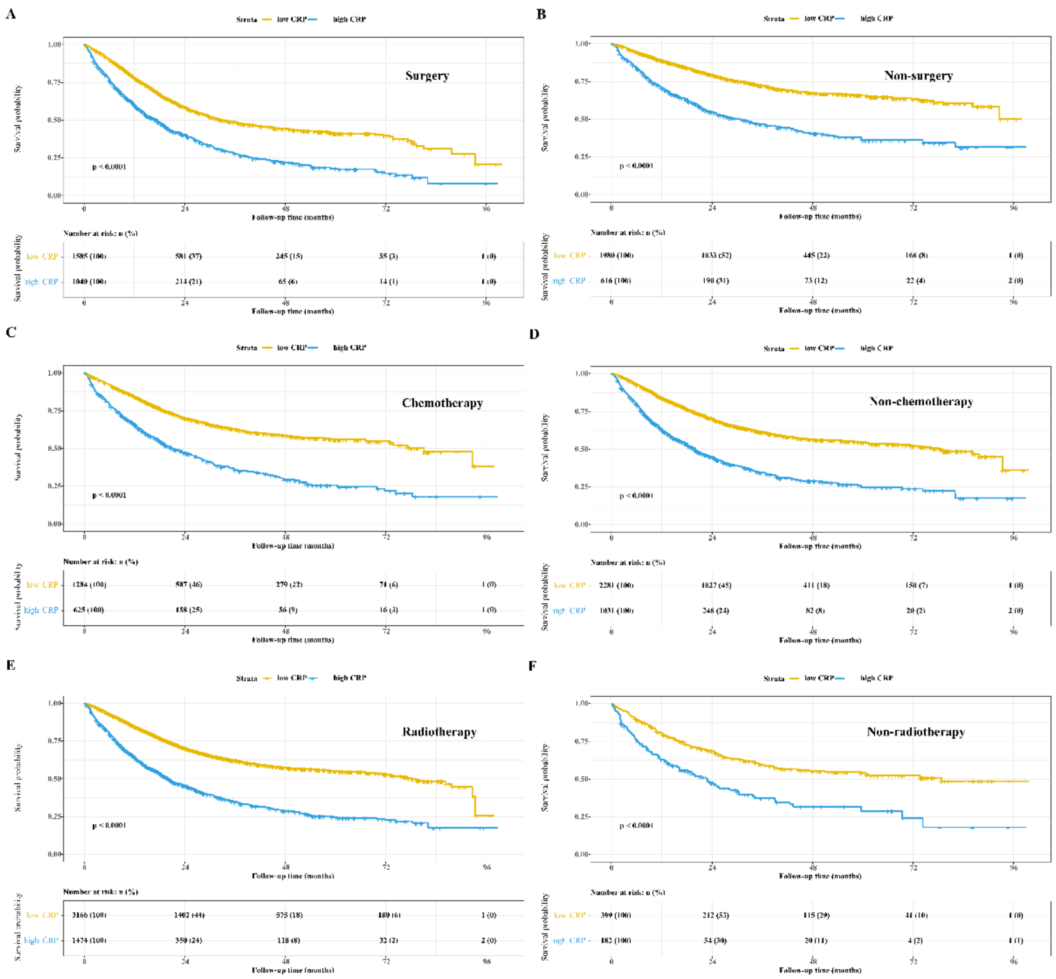

Additional file 5 The Kaplan-Meier survival curves of CRP in different subgroups. (A) non-surgery patients; (B) surgery patients; (C) non-chemotherapy patients; (D) chemotherapy patients; (E) non-radiotherapy patients; (F) radiotherapy patients.

Notes: CRP: C-reactive protein.
