# Supplementary material for: Prognostic importance of systemic inflammation and insulin resistance in patients with cancer: a prospective multicenter study
Source: BMC Cancer. 2022 Jun 25;22:700. doi: 10.1186/s12885-022-09752-5 (PMC9233357; doi:10.1186/s12885-022-09752-5)
Supplement: Supplementary file 8 — Additional file 8. The Kaplan-Meier survival curves of LHR in different subgroups. (A) BMI<18.5; (B) BMI:18.5-24; (C) BMI: 24-28; (D) BMI>28; (E) TNM stage I; (F) TNM stage II; (G) TNM stage III; (H) TNM stage IV. Notes: CRP: C-reactive protein. [file 12885_2022_9752_MOESM8_ESM.pdf]

Additional file 8

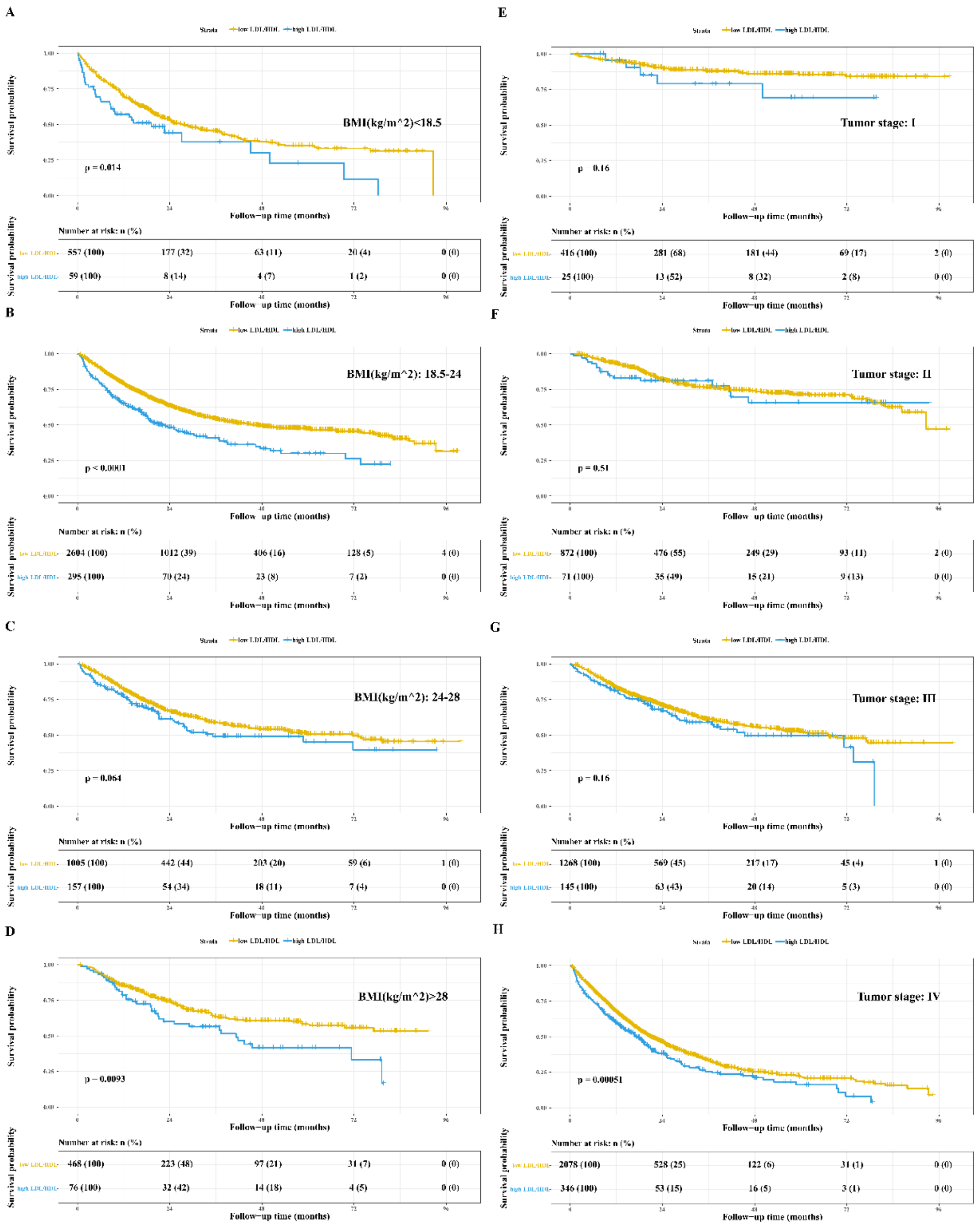

Additional file 8 The Kaplan-Meier survival curves of LHR in different subgroups. (A) BMI<18.5; (B) BMI:18.5-24; (C) BMI: 24-28; (D) BMI>28; (E) TNM stage I; (F) TNM stage II; (G) TNM stage III; (H) TNM stage IV.

Notes: LHR: LDL-c/HDL-c ratio; HDL-c: high-density lipoprotein cholesterol; LDL-c: low-density lipoprotein cholesterol.
