# Supplementary material for: Prognostic importance of systemic inflammation and insulin resistance in patients with cancer: a prospective multicenter study
Source: BMC Cancer. 2022 Jun 25;22:700. doi: 10.1186/s12885-022-09752-5 (PMC9233357; doi:10.1186/s12885-022-09752-5)
Supplement: Supplementary file 9 — Additional file 9. [file 12885_2022_9752_MOESM9_ESM.pdf]

Additional file 9 Sensitivity analysis*

Variables	OS (model 0)		OS (model 4)	
	Crude HR (95%CI)	Crude *P*	Adjusted HR (95%CI)	Adjusted *P*
CRP				
As continuous (per SD)	1.22 (1.17-1.27)	<0.001	1.11 (1.06-1.16)	<0.001
as binary				
CRP≤10				
CRP>10	2.25 (2.05-2.48)	<0.001	1.47 (1.33-1.62)	<0.001
as quartile				
Q1(<2.60)				
Q2(2.60-3.71)	1.49 (1.20-1.84)	<0.001	1.33 (1.08-1.66)	0.009
Q3 (3.71-15.80)	1.96 (1.61-2.38)	<0.001	1.58 (1.30-1.93)	<0.001
Q4 (>15.80)	2.62 (2.17-3.16)	<0.001	1.72 (1.41-2.10)	<0.001
p for trend		<0.001		<0.001
LHR				
As continuous (per SD)	1.15 (1.11-1.20)	<0.001	1.06 (1.02-1.10)	0.002
as binary				
LHR≤3.56				
LHR>3.56	1.92 (1.69-2.17)	<0.001	1.55 (1.36-1.76)	<0.001
as quartile				
Q1(<1.81)				
Q2(1.81-2.33)	0.97 (0.81-1.16)	0.766	0.98 (0.82-1.18)	0.829
Q3 (2.33-2.94)	0.96 (0.81-1.13)	0.624	0.99 (0.83-1.17)	0.895
Q4 (>2.94)	1.02 (0.82-1.26)	0.858	1.10 (0.88-1.37)	0.408
p for trend		0.927		0.576

Notes: * The sensitivity analysis was to exclude patients who died within 6 months. LHR: LDL-c/HDL-c ratio; HDL-c: high-density lipoprotein cholesterol; LDL-c: low-density lipoprotein cholesterol; HR, hazards ratio; CI, confidence interval; BMI: body mass index; KPS, karnofsky performance status.

Model 0: Unadjusted.

Model 4: Adjusted for age, sex, tumor stage, BMI, tumor types, KPS, surgery, chemotherapy, radiotherapy, smoking, alcohol, nutritional intervention, diabetes, hypertension, and coronary heart disease.
