# Supplementary material for: Prognostic importance of systemic inflammation and insulin resistance in patients with cancer: a prospective multicenter study
Source: BMC Cancer. 2022 Jun 25;22:700. doi: 10.1186/s12885-022-09752-5 (PMC9233357; doi:10.1186/s12885-022-09752-5)
Supplement: Supplementary file 10 — Additional file 10. [file 12885_2022_9752_MOESM10_ESM.zip › additional file 10 continuation.pdf]

disease.
