# Supplementary material for: Prognostic importance of systemic inflammation and insulin resistance in patients with cancer: a prospective multicenter study
Source: BMC Cancer. 2022 Jun 25;22:700. doi: 10.1186/s12885-022-09752-5 (PMC9233357; doi:10.1186/s12885-022-09752-5)
Supplement: Supplementary file 10 — Additional file 10. [file 12885_2022_9752_MOESM10_ESM.zip › additional file 10.pdf]

Additional file 10 Univariate and multivariate analysis of CRP in different tumor types

Variables	OS (model 0)		OS (model 4)	
	Crude HR (95%CI)	Crude *P*	Adjusted HR (95%CI)	Adjusted *P*
Lung cancer				
CRP≤10	1		1	
CRP>10	1.74 (1.53-1.99)	<0.001	1.27 (1.11-1.46)	0.001
Gastrointestinal cancer				
Gastric cancer				
CRP≤10	1		1	
CRP>10	1.72 (1.36-2.18)	<0.001	1.37 (1.07-1.75)	0.014
Colorectal cancer				
CRP≤10	1		1	
CRP>10	3.28 (2.60-4.12)	<0.001	2.42 (1.89-3.10)	<0.001
Esophageal cancer				
CRP≤10	1		1	
CRP>10	1.81 (1.31-2.49)	<0.001	1.54 (1.09-2.17)	0.013
Other gastrointestinal cancer				
CRP≤10	1		1	
CRP>10	2.50 (1.90-3.30)	<0.001	1.70 (1.24-2.32)	0.001
Breast cancer				
CRP≤10	1		1	
CRP>10	2.87 (1.55-5.32)	0.001	1.64 (0.86-3.15)	0.135
Female reproductive cancer				
CRP≤10	1		1	
CRP>10	1.52 (0.91-2.55)	0.111	1.05 (0.60-1.86)	0.856
Urological cancer				
CRP≤10	1		1	
CRP>10	3.59 (1.98-6.51)	<0.001	3.88 (1.81-8.32)	0.001
Nasopharyngeal cancer				
CRP≤10	1		1	
CRP>10	6.38 (2.56-15.85)	<0.001	10.53 (2.31-48.11)	0.002
Other cancer subtypes				
CRP≤10	1		1	
CRP>10	2.29 (1.29-4.09)	0.005	2.01 (1.06-3.84)	0.033

Notes: CRP: C-reactive protein; HR, hazards ratio; CI, confidence interval; BMI: body mass index; KPS, karnofsky performance status.

Model 0: Unadjusted.

Model 4: Adjusted for age, sex, tumor stage, BMI, tumor types, KPS, surgery, chemotherapy, radiotherapy, smoking, alcohol, nutritional intervention, diabetes, hypertension, and coronary heart
