# Supplementary material for: Prognostic importance of systemic inflammation and insulin resistance in patients with cancer: a prospective multicenter study
Source: BMC Cancer. 2022 Jun 25;22:700. doi: 10.1186/s12885-022-09752-5 (PMC9233357; doi:10.1186/s12885-022-09752-5)
Supplement: Supplementary file 11 — Additional file 11. [file 12885_2022_9752_MOESM11_ESM.zip › additional file 11.pdf]

Additional file 11 Univariate and multivariate analysis of LHR in different tumor types

Variables	OS (model 0)		OS (model 4)	
	Crude HR (95%CI)	Crude *P*	Adjusted HR (95%CI)	Adjusted *P*
Lung cancer				
LHR≤3.56	1		1	
LHR>3.56	1.39 (1.13-1.71)	0.002	1.08 (0.88-1.34)	0.459
Gastrointestinal cancer				
Gastric cancer				
LHR≤3.56	1		1	
LHR>3.56	1.67 (1.18-2.35)	0.003	1.48 (1.03-2.13)	0.036
Colorectal cancer				
LHR≤3.56	1		1	
LHR>3.56	1.77 (1.33-2.35)	<0.001	1.57 (1.17-2.11)	0.003
Esophageal cancer				
LHR≤3.56	1		1	
LHR>3.56	1.38 (0.88-2.17)	0.166	1.35 (0.82-2.21)	0.240
Other gastrointestinal cancer				
LHR≤3.56	1		1	
LHR>3.56	1.48 (1.01-2.16)	0.046	0.96 (0.62-1.47)	0. 837
Breast cancer				
LHR≤3.56	1		1	
LHR>3.56	2.54 (1.09-5.92)	0.030	2.08 (0.85-5.07)	0.108
Female reproductive cancer				
LHR≤3.56	1		1	
LHR>3.56	0.85 (0.31-2.34)	0.747	0.78 (0.27-2.24)	0.645
Urological cancer				
LHR≤3.56	1		1	
LHR>3.56	1.39 (0.65-2.97)	0.397	0.93 (0.36-2.40)	0.874
Nasopharyngeal cancer				
LHR≤3.56	1		1	
LHR>3.56	0.74 (0.17-3.23)	0.693	0.77 (0.14-4.34)	0. 765
Other cancer subtypes				
LHR≤3.56	1		1	
LHR>3.56	0.99 (0.49-1.99)	0.978	0.85 (0.40-1.81)	0.675

Notes: LHR: LDL-c/HDL-c ratio; HDL-c: high-density lipoprotein cholesterol; LDL-c: low-density lipoprotein cholesterol; HR, hazards ratio; CI, confidence interval; BMI: body mass index; KPS, karnofsky performance status.

Model 0: Unadjusted.

Model 4: Adjusted for age, sex, tumor stage, BMI, tumor types, KPS, surgery, chemotherapy, radiotherapy, smoking, alcohol, nutritional intervention, diabetes, hypertension, and coronary heart
