# Supplementary material for: Prognostic importance of systemic inflammation and insulin resistance in patients with cancer: a prospective multicenter study
Source: BMC Cancer. 2022 Jun 25;22:700. doi: 10.1186/s12885-022-09752-5 (PMC9233357; doi:10.1186/s12885-022-09752-5)
Supplement: Supplementary file 12 — Additional file 12. The distribution of CRP between high LHR and low LHR. Notes: CRP: C-reactive protein; LHR: LDL-c/HDL-c ratio; HDL-c: high-density lipoprotein cholesterol; LDL-c: low-density lipoprotein cholesterol. [file 12885_2022_9752_MOESM12_ESM.pdf]

Additional file 12

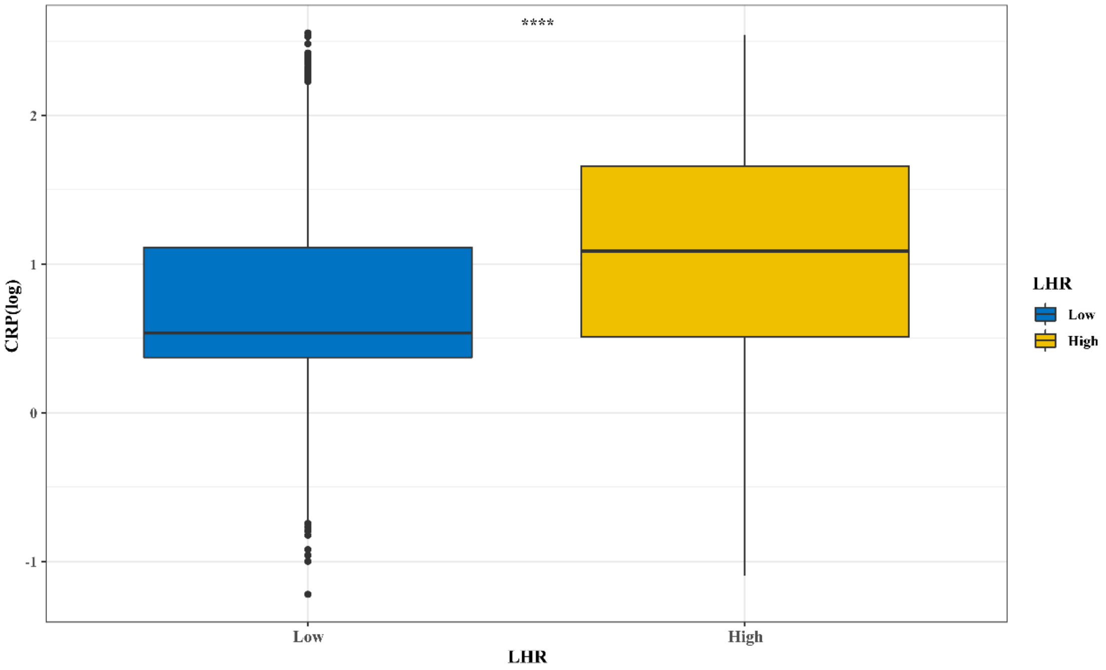

Additional file 12 The distribution of CRP between high LHR and low LHR.

Notes: CRP: C-reactive protein; LHR: LDL-c/HDL-c ratio; HDL-c: high-density lipoprotein cholesterol; LDL-c: low-density lipoprotein cholesterol.
